# Ageing in *Drosophila*: The role of the insulin/Igf and TOR signalling network

**DOI:** 10.1016/j.exger.2010.09.003

**Published:** 2011-05

**Authors:** Linda Partridge, Nazif Alic, Ivana Bjedov, Matt D.W. Piper

**Affiliations:** a Institute of Healthy Ageing, and G.E.E., UCL, Gower Street, London, WC1E 6BT, UK; b Max Planck Institute for Biology of Ageing, Gleueler Straße 50a, 50931 Köln, Germany

**Keywords:** Aging, Drosophila, Insulin/Igf signalling, TOR, Dietary restriction

## Abstract

A remarkable discovery of recent years is that, despite the complexity of ageing, simple genetic interventions can increase lifespan and improve health during ageing in laboratory animals. The pathways involved have often proved to sense nutrients and to match costly activities of organisms, such as growth, metabolism and reproduction, to nutrient status. For instance, the insulin/insulin-like growth factor and Target of Rapamycin signalling network has proved to play a function in ageing, from yeast to mammals, seemingly including humans. In the fruit fly *Drosophila*, altered activity of several components of this network can increase lifespan and improve locomotor and cardiac function during ageing. The fly brain, fat body (equivalent of mammalian liver and white adipose tissue) and the germ line are important in determination of lifespan, with considerable communication between different tissues. Cellular detoxification pathways, increased autophagy and altered protein synthesis have all been implicated in increased lifespan from reduced IIS/TOR activity, with the role of defence against oxidative stress unresolved. Reduced IIS/TOR signalling can alter or block the response of lifespan to dietary restriction. Reduced IIS can act acutely to lower death rate, implying that it may ameliorate the effects of ageing-related damage, rather than preventing it.

## Research Highlights

► Mutations in nutrient sensing pathways extend lifespan in many organisms, including Drosophila. ► Insulin/insulin-like and TOR signalling are two such pathways. ► Interactions between nutrition, signalling and possible mechanisms are discussed.

## 1 Introduction

**Fig. 1:**
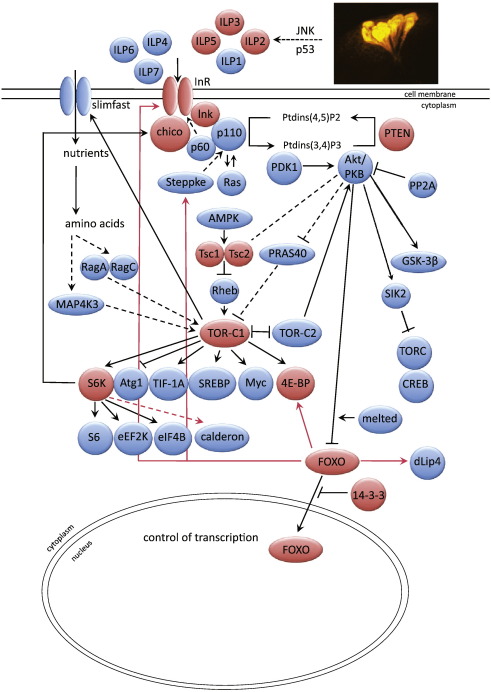
The IIS/TOR signalling network in *Drosophila*, adapted from Teleman (2010). Those components that have been experimentally demonstrated to play a role in fly lifespan are coloured red. Arrows indicate activation, but not necessarily direct physical interactions, bar-ended lines indicate inhibitory interactions. Broken lines indicate indirect or less certain interactions. Red arrows indicate transcriptional regulation.

A breakthrough in research into ageing has come from the discovery of single gene mutations that extend the healthy lifespan of laboratory animals. Evolutionary conservation of the underlying mechanisms is at present clearest for the classic nutrient-sensing pathways, the insulin/insulin-like growth factor (Igf) and Target of Rapamycin (TOR) network (Evans et al., 2011; Fontana et al., 2010; Kenyon, 2010; Partridge, 2010). Reduced insulin/Igf-like signalling (IIS) was first shown to extend lifespan in the nematode worm *Caenorhabditis elegans*, as a result of direct genetic screens for lifespan-extending mutations (Kenyon, 2010; Partridge, 2010). The fruit fly *Drosophila* provided the initial evidence for evolutionary conservation of the anti-ageing effect of reduced IIS (Clancy et al., 2001; Tatar et al., 2001). Reduced activity of the amino acid sensing TOR pathway, which interacts extensively with IIS (Fig. 1), was also initially shown to extend the lifespan in *C. elegans* (Vellai et al., 2003), again with evolutionary conservation first demonstrated in *Drosophila* (Kapahi et al., 2004), and subsequently in yeast (reviewed in (Kenyon, 2010)). Importantly, reduced activity of both of these pathways, whether through genetic manipulation or chemical inhibition, can extend the lifespan in mice (reviewed in (Fontana et al., 2010; Kenyon, 2010), and can produce a remarkably broad-spectrum improvement in health during middle and old age (Selman et al., 2008, 2009). Seven independent population-genetic studies of lifespan in humans have also pointed to an association with genetic variation in Foxo3a, a human orthologue of the key IIS effector in *C. elegans*, the forkhead transcription factor DAF-16 (reviewed in (Kenyon, 2010)). We can thus use simpler and shorter-lived laboratory model organisms, including *Drosophila*, to understand at least certain aspects of human ageing.

The fruit fly has both strengths and weaknesses for ageing research. On one hand, it has the advantages of a well-established model organism, including powerful genetic and genomic reagents and methodologies and increasingly well understood physiology and behaviour. On the other hand, much that is known about *Drosophila* has been learned from embryos, larvae and the process of metamorphosis. The adult fly, the subject of studies of longevity, is significantly less well known generally and the effects of the IIS/TOR network are much less thoroughly explored. However, *Drosophila* is more mammal-like than *C. elegans*, with a well-differentiated brain and complex behaviours, a heart, homologues of kidneys (the Malpighian tubules) and dioecy (females and males). It also has powerful systems for gene knock-out and targeted mutagenesis, it is an excellent organism for work on demography and many genetic models of human ageing-related diseases have been developed.

The aim of this review is to synthesise what is known of the role of the IIS/TOR network in the determination of *Drosophila* lifespan, particularly in respect of those aspects that are strong candidates for conservation in mammals.

## 2 IIS/TOR signalling and lifespan in flies

**Fig. 2:**
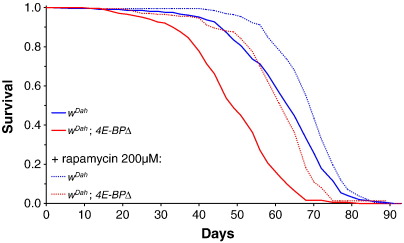
Effect of rapamycin on lifespan. Rapamycin treatment extends lifespan of *w*^*Dah*^ females. Compared to flies on control food (0 μM rapamycin), flies on 200 μM rapamycin food have increased median lifespan (*p* < 0.0001, log-rank test). In *w*^*Dah*^ background, rapamycin also extends lifespan of *4E-BPΔ* mutant female flies (*p* < 0.0001, log-rank test). 4E-BP improves survival, as *4E-BPΔ* flies live shorter than the control flies on standard food (*p* < 0.0001, log-rank test).

The most direct indication that an intervention slows ageing is to demonstrate that it extends organismal lifespan. A diagram of the signalling role of the insulin/Igf/TOR network in growth and metabolism in *Drosophila* (modified from Teleman, 2010) is shown in Fig. 1, with those components and links demonstrated to increase adult lifespan highlighted. Reduced expression of *Drosophila* insulin-like peptides (*dilp*s) (Gronke et al., 2010), of the insulin receptor (Tatar et al., 2001; Ikeya, et al., 2009 #174) and receptor substrates *chico* (Clancy et al., 2001) and *Lnk* (Slack et al., 2010) extends lifespan, as does increased expression of the negative pathway regulator PTEN (Hwangbo et al., 2004). In *C. elegans* the forkhead transcription factor DAF-16 is negatively regulated by IIS and it is essential for extension of lifespan by reduced IIS. It is not yet known if its *Drosophila* orthologue dFOXO is also required for IIS to extend lifespan, but over-expression of dFOXO itself extends lifespan (Giannakou et al., 2004; Hwangbo et al., 2004) as does reduced expression of its negative regulator 14-3-3ε (Nielsen et al., 2008). Reduced activity of the TOR pathway through modulation of several genes, including dTsc1, dTsc2, dTOR and dS6K also extends lifespan (Kapahi et al., 2004), as does inhibition of TORC1 by rapamycin (Bjedov et al., 2010). Although the lifespan of flies null for the inhibitor of cap-dependent translation 4E-BP that lies downstream of TORC1 can also fail to respond to rapamycin (Bjedov et al., 2010), lifespan of these mutants can be increased in at least some genetic backgrounds (Fig. 2), implying that 4E-BP is not required for inhibition of TORC1 to extend lifespan. These experiments were conducted in the outbred and long-lived white Dahomey genetic background, where the null mutation for 4E-BP itself reduced lifespan only moderately, and the previous failure to extend lifespan by rapamycin in this mutant may have been because the genetic background used rendered the mutant too generally sick to respond to the drug.

Many wild populations and laboratory stocks of *Drosophila* are host to the endosymbiotic bacterium *Wolbachia pipientis*. Removal of *Wolbachia* from wild type stocks by antibiotic treatment can increase lifespan, suggesting that the bacterium can be life-shortening (Min and Benzer, 1997). *Wolbachia* also shows intriguing interactions with IIS. Removal of the bacterium can enhance IIS mutant phenotypes (Ikeya et al., 2009), implying that the bacterium may increase IIS. Furthermore, some mutants that reduce IIS extend lifespan only in the presence of *Wolbachia* (Gronke et al., 2010), possibly because the bacterium can attenuate deleterious effects of IIS down-regulation. Lifespan may be particularly sensitive to changes in IIS or there may be more specific interactions between *Wolbachia* and IIS in *Drosophila*.

## 3 Which tissues mediate extension of lifespan?

The two main tissues so far implicated in extension of lifespan from reduced insulin/TOR signalling are the nervous system and the fat body, the fly equivalent of mammalian white adipose tissue and liver, with documented cross-talk between these two tissues. Communication between the germ line and systemic IIS is important in determination of lifespan.

In adult *Drosophila dilp*s 2, 3 and 5 are produced in a set of median neurosecretory cells (MNC) in the brain. Targeted ablation of these cells late in larval development extends lifespan (Broughton et al., 2005), as does deletion of the genes encoding these 3 DILPs (Gronke et al., 2010). There is extensive feedback in the expression of these 3 brain *dilps*, with positive regulation of *dilp*s 2 and 5 by *dilp* 3, and several compensatory interactions between them (Gronke et al., 2010). There is also cross-talk between the *dilps* in the brain and *dilp6* in the fat body. When *dilps* 2, 3, and 5 genes are deleted, there is a substantial compensatory increase in the expression of *dilp6* in the fat body, although deletion of *dilp6* does not alter the expression of the brain *dilps*, and nor does it result in an increase in lifespan. The triple *dilp 2*, 3 and 5 deletion mutant and the single *dilp*6 mutant are both viable, but deletion of all 4 of these *dilps* is lethal (Gronke et al., 2010), implying functional redundancy between them.

The *dilp*-producing MNC in the brain act as an important site for integration of external inputs to IIS, including stress and nutrient status. For instance, Jun-N-terminal kinase (JNK) is activated in response to a variety of stresses, and flies with genetically increased JNK activity show increased resistance to paraquat and extended lifespan (Wang et al., 2003). These changes are mediated by the fly forkhead transcription factor dFOXO, and at least part of the mechanism may be through reduced systemic IIS, because activation of JNK signalling specifically in the brain MNC reduces transcript levels of *dilps* 2 and 5 (Karpac et al., 2009; Wang et al., 2005). Similarly, expression of a dominant-negative form of the tumour suppressor *Dmp53* in these cells both extends lifespan and reduces transcript level of *dilp2* (Bauer et al., 2007), which when deleted extends fly lifespan (Gronke et al., 2010). Dietary restriction (DR) in *Drosophila*, which extends fly lifespan, results in reduced levels of *dilp5* transcript in the MNC (Min et al., 2008), although deletion of the *dilp5* gene alone does not alter the response to DR, possibly because of increased compensatory expression of *dilps* 3 (Gronke et al., 2010).

Temporally- and spatially-controlled over-expression of the forkhead transcription factor dFOXO in the fat body of the adult fly extends lifespan (Giannakou et al., 2004; Hwangbo et al., 2004), demonstrating both that the developmental effects of reduced IIS can be uncoupled from its effects on lifespan and a role for the fat body in the control of lifespan. The mechanisms involved require further elucidation. Expression of *dilp*2 in the brain has been reported to decrease upon over-expression of *dfoxo* in pericerebral fat body and to be associated with a decrease in insulin signalling in the abdominal fat body (Hwangbo et al., 2004).

In *C. elegans*, ablation of the germ line increases lifespan, while ablation of the germ line and somatic gonad together leaves lifespan unaffected, implying that signals from the germ line shorten lifespan, and that these signals are transduced by the somatic gonad (Arantes-Oliveira et al., 2002). Removal of the germ line activates the worm forkhead transcription factor *daf-16*, and this gene is necessary for the extension of lifespan by germ line removal. Interestingly, the negative effect of the germ line on lifespan appears to be conserved in *Drosophila*. Although the life-long absence of the germ line has no effect on lifespan (Barnes et al., 2006), loss of germ cells late in development or in the adult extends lifespan of both sexes. This is accompanied by apparent peripheral insulin resistance and a, possibly compensatory, increase in expression of *dilps* 2, 3 and 5 in the brain (Flatt et al., 2008).

Understanding the mechanistic basis of the interactions between these different tissues will be an important step in understanding the signalling mechanisms at work in the extension of lifespan.

## 4 What cellular biochemical processes are altered to increase lifespan?

Increased lifespan could be a consequence of improved defences against processes leading to loss of function and death, reduction in activities that feed into these destructive processes, or both. An unbiased approach to this question, using gene expression profiles from long-lived, IIS mutant worms, flies and mice, showed that cellular detoxification pathways were strongly up-regulated in all 3 organisms (McElwee et al., 2007). Indeed, experimentally increasing the activity of a key transcriptional regulator of this pathway can increase the lifespan in *Drosophila* (Sykiotis and Bohmann, 2008). Detoxification and elimination of lipophilic endobiotics and xenobiotics may therefore be important processes for protection against the effects of ageing.

Increased lifespan as a result of decreased IIS signalling has often been associated with increased resistance to oxidative stress, and *Drosophila* is no exception. However experiments aimed at directly testing for a role of reactive oxygen species or oxidative stress in ageing have met with mixed results (Piper et al., 2008). This may be in part because the idea is difficult to test cleanly, because free radicals not only generate damage to macromolecules, but also affect other processes, such as cellular signalling.

Rapamycin increases *Drosophila* lifespan through inhibition of TORC1, and it also decreases levels of protein translation and induces autophagy. Both of these effects of rapamycin may be causal in the extension of lifespan, because constitutive activation of the S6 kinase and blocking of autophagy block the increase in longevity (Bjedov et al., 2010), although the precise mechanisms require further study.

## 5 Does the IIS/TOR network mediate the effects of dietary restriction?

Dietary restriction (DR) is a reduction in food intake that falls short of malnutrition or starvation, and it has been shown to increase lifespan and reduce fecundity in a wide range of organisms (Mair and Dillin, 2008). DR in *Drosophila* can be implemented in different ways, such as manipulation of the presence of live yeast paste, of the concentration of the whole diet or of specific nutrients in the diet. In addition, a variety of diets have been used for DR. The IIS/TOR network may well respond differently to these specific interventions. Flies do not show compensatory feeding in response to dilution of at least some ingredients in the diet during DR (Wong et al., 2008; Grandison et al., 2009).

**Fig. 3:**
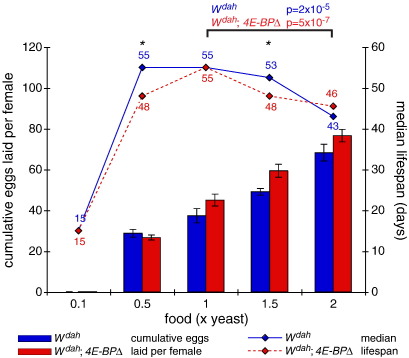
DR in *4E-BPΔ* and wild-type females. DR extends lifespan in both wild-type and *4E-BPΔ w*^*Dah*^ female flies. Median lifespan values (diamonds) and cumulative number of eggs laid per female (bars) are plotted for the wild-type (blue) or *4E-BPΔ* (red) female flies as a function of the yeast concentration in the food. Asterisk denotes the food concentration where a significant difference for lifespan was detected between the wild-type and *4E-BPΔ* flies by Log-rank test (*p* < 0.05). The same test was used to determine the difference in lifespan between 1× and 2× yeast for each genotype and the *p* values are given above the graph.

Most work on the role of the IIS/TOR network has examined the effect of mutants on the response to DR. Initially, both loss of *chico* (Clancy et al., 2002) and reduced TOR activity (Kapahi et al., 2004) caused lifespan to peak at a higher food concentration than wild type DR, with a greater extension of lifespan by these mutants at higher levels of food intake. These results imply that reduced signalling activity may induce a DR-like state. The downstream IIS/TOR effector 4E-BP can extend lifespan when over-expressed, and is required for the response to DR on food containing yeast extract (Zid et al., 2009). However flies null for 4E-BP respond normally to DR using a broader range of dilutions of whole-cell yeast lysate (Fig. 3), indicating that the role of 4E-BP may therefore depend upon the type of DR.

In *C. elegans*, the role of IIS in mediating the response to an environmental intervention is generally assessed by the effect of reduction in the function of *daf-16*, which is required for the increase in lifespan in response to reduced IIS. In contrast, *daf-16* is not required for the response of lifespan to most forms of DR in C. elegans (Mair and Dillin, 2008), which has led to the conclusion that these two interventions extend lifespan by different mechanisms. Flies that are null for *dfoxo* are short-lived, but they also respond normally to at least one form of DR (Giannakou et al., 2008; Min et al., 2008). However, flies with over-expression of *dfoxo* in the fat body behave like *chico* and TOR mutants, with greater extension of lifespan relative to controls at higher food intake levels, and a peak in lifespan at higher food concentrations (Giannakou et al., 2008). These findings imply that *dfoxo* may normally play a role in the response to DR, but that other mechanisms can compensate for the loss of *dfoxo*.

Loss of the *dilp*-producing MNC in the brain (Broughton et al., 2010), deletion of *dilps* 2, 3 and 5 (Gronke et al., 2010) and over-expression of a dominant-negative form of the insulin receptor (Grandison et al., 2009) all extend lifespan at higher food concentrations, and largely abolish the effect of food intake on both lifespan and fecundity, while rapamycin has similar effects on the response of lifespan to DR (Bjedov et al., 2010). These interactions of IIS and TOR signalling with DR imply that the signalling network has a role in mediating the responses to DR. However, in general the interpretation of this kind of epistasis analysis can be tricky (Gems et al., 2002), and it will be important to establish the exact nutritional and molecular mediators of the normal response to DR in the fly.

## 6 Does reduced IIS/TOR signalling slow ageing in *Drosophila*?

Although increased lifespan is an important output, it might be expected that an intervention would also improve health and function at later ages if the ageing process was ameliorated. Reduced activity of the IIS/TOR network can improve some aspects of function during ageing, and can also reduce the pathology associated with genetic models of specific ageing-related diseases, but much more work is needed to understand the generality of these effects and the mechanisms mediating them.

Fecundity drops with age in female flies, as a result of loss of germ cells, because of failure of the germ cell niche (Pan et al., 2007). Reduced activity of the IIS/TOR network often decreases female fecundity, and IIS/TOR signalling plays an important role in growth, proliferation and survival of the ovarian stem cells, both germ line and somatic (Hsu et al., 2008; LaFever and Drummond-Barbosa, 2005; LaFever et al., 2010; Sun et al., 2010). Interestingly, female mutants for the insulin receptor *dInR* or for *chico*, which are long-lived, show more rapid loss of germ line stem cells with age, and over-expression of *dilp2* in ovarian somatic cells counteracts both the age-related loss in wild type flies and the accelerated loss in IIS mutants (Hsu and Drummond-Barbosa, 2009), implying that IIS may reduce reproductive ageing. Surprisingly, and in contrast, dietary restriction (DR), the effects of which may be mediated at least in part by reduced activity of the IIS/TOR network, enhances germ line stem cell maintenance with age (Mair et al., 2010). It will be important to understand the mechanisms by which these two, apparently related, interventions have opposite effects on reproductive ageing.

Both reduced IIS (Wessells et al., 2004) and TOR signalling (Luong et al., 2006) can protect against loss of cardiac function during ageing, and this protection is mediated through 4E-BP (Wessells et al., 2009). Locomotor performance, including negative geotaxis, also shows a marked age-related decline in flies, and a genetic screen for delayed decline revealed that mutations in *chico*, the phosphoinositide-dependent kinase 1 (pdk-1), the catalytic subunit of the PI3 kinase (Dp110) and the protein kinase B (Akt) all delayed the loss of negative geotaxis (Jones et al., 2009).

As yet, little work has been done in *Drosophila* on the interaction between the IIS/TOR signalling network and genetic models of specific ageing-related diseases, but where it has, the focus has been mainly on fly models of neurodegeneration. For instance, fly models of Alzheimer's Disease (AD) have investigated mechanisms of toxicity of the Amyloid Precursor Protein and its derivatives, and of the microtubule-associated protein tau, both strongly implicated in the aetiology of human AD. Similarly, alpha-synuclein is strongly implicated in age-related, sporadic, Parkinson's Disease in humans, and fly models that over-express this protein have been developed . Reduced activity of components of the IIS/TOR network can reduce the neurotoxicity from all of these proteins and from genetic models of other types of neurodegenerative disease (reviewed in Hirth, 2010 #202). However, so far investigation of the mechanisms at work has barely scratched the surface. For instance, it is quite unclear what specific modulations of IIS/TOR signalling are required to produce neuroprotection, whether these modulations are the same for protection against different forms of neurotoxicity or whether there is an inevitable association between neuroprotection and extension of lifespan.

Demographic analysis provides another avenue for addressing the issue of whether lifespan is increased through a reduced rate of ageing. In *Drosophila*, rather than slowing the accumulation of irreversible damage, DR acts entirely acutely to reduce the death rate of the flies. If the diet of the flies is switched to or from DR, they immediately adopt the death rates of flies of the same age that are kept permanently in the new dietary regime. There is thus no effect of dietary history on death rate, suggesting that the flies are acutely protected against a diet-related risk of dying with ageing, but that their underlying rate of ageing has not been slowed down. This contrasts sharply with the effects of temperature, where thermal history is the sole determinant of likelihood of death (Mair et al., 2003). These two interventions, diet and temperature, are also associated with very different forms of molecular damage (Jacobson et al., 2010). Use of an inducible system for gene expression allows switching of IIS status part-way through adulthood and, as for DR, up to the age of one month, reduced IIS acts at least partly acutely to lower death rate (Giannakou et al., 2007). Understanding how the molecular mechanisms can act acutely to protect against loss of function and death will be important in identifying potential drug targets.

## 7 Conclusions and future directions

Although an enormous amount of progress has been made in the few years since the role of these nutrient-sensing pathways in ageing was discovered, we have barely begun to understand the mechanisms at work. These pathways are highly pleiotropic in their effects, with influences on growth, fecundity, stress resistance and metabolism, as well as on survival. It is not yet clear to what extent the less desirable effects of reducing their activity can be separated from the beneficial effects on lifespan, nor have possible negative side-effects, for instance on wound healing and immunity, been fully explored. Understanding these issues will be vital to pinpoint the levels in the signalling network where intervention might be beneficial and for identifying potential drug targets to improve health during ageing. Information on the timing of the effects on survival, and specifically whether they are acute, will also be important for understanding the stage(s) during adulthood at which intervention could be useful. The fly is an excellent model organism for work on genetic models of ageing-related disease and the role of ageing as a risk factor, an area that has as yet been hardly touched.

## References

[bb0005] Arantes-Oliveira N., Apfeld J., Dillin A., Kenyon C. (2002). Regulation of life-span by germ-line stem cells in *Caenorhabditis elegans*. Science.

[bb0010] Barnes A.I., Boone J.M., Jacobson J., Partridge L., Chapman T. (2006). No extension of lifespan by ablation of germ line in *Drosophila*. Proc. R. Soc. Lond. B Biol. Sci..

[bb0015] Bauer J.H., Chang C., Morris S.N., Hozier S., Andersen S., Waitzman J.S., Helfand S.L. (2007). Expression of dominant-negative Dmp53 in the adult fly brain inhibits insulin signaling. Proc. Natl. Acad. Sci. USA.

[bb0020] Bjedov I., Toivonen J.M., Kerr F., Slack C., Jacobson J., Foley A., Partridge L. (2010). Mechanisms of life span extension by rapamycin in the fruit fly *Drosophila melanogaster*. Cell Metab..

[bb0025] Broughton S.J., Piper M.D., Ikeya T., Bass T.M., Jacobson J., Driege Y., Martinez P., Hafen E., Withers D.J., Leevers S.J., Partridge L. (2005). Longer lifespan, altered metabolism, and stress resistance in *Drosophila* from ablation of cells making insulin-like ligands. Proc. Natl. Acad. Sci. USA.

[bb0030] Broughton S.J., Slack C., Alic N., Metaxakis A., Bass T.M., Driege Y., Partridge L. (2010). DILP-producing median neurosecretory cells in the *Drosophila* brain mediate the response of lifespan to nutrition. Aging Cell.

[bb0035] Clancy D.J., Gems D., Harshman L.G., Oldham S., Stocker H., Hafen E., Leevers S.J., Partridge L. (2001). Extension of life-span by loss of CHICO, a *Drosophila* insulin receptor substrate protein. Science.

[bb0040] Clancy D.J., Gems D., Hafen E., Leevers S.J., Partridge L. (2002). Dietary restriction in long-lived dwarf flies. Science.

[bb0045] Evans D.S., Kapahi P., Hsueh W.C., Kockel L. (2011). TOR signaling never gets old: aging, longevity and TORC1 activity. Ageing Res. Rev..

[bb0050] Flatt T., Min K.J., D'Alterio C., Villa-Cuesta E., Cumbers J., Lehmann R., Jones D.L., Tatar M. (2008). *Drosophila* germ-line modulation of insulin signaling and lifespan. Proc. Natl. Acad. Sci. USA.

[bb0055] Fontana L., Partridge L., Longo V.D. (2010). Extending healthy life span-from yeast to humans. Science.

[bb0060] Gems D., Pletcher S., Partridge L. (2002). Interpreting interactions between treatments that slow aging. Aging Cell.

[bb0065] Giannakou M.E., Goss M., Junger M.A., Hafen E., Leevers S.J., Partridge L. (2004). Long-lived *Drosophila* with overexpressed dFOXO in adult fat body. Science.

[bb0070] Giannakou M.E., Goss M., Jacobson J., Vinti G., Leevers S.J., Partridge L. (2007). Dynamics of the action of dFOXO on adult mortality in *Drosophila*. Aging Cell.

[bb0075] Giannakou M.E., Goss M., Partridge L. (2008). Role of dFOXO in lifespan extension by dietary restriction in *Drosophila* melanogaster: not required, but its activity modulates the response. Aging Cell.

[bb0080] Grandison R.C., Piper M.D.W., Partridge L. (2009). Amino acid imbalance and not resource reallocation explains extension of lifespan by dietary restriction in *Drosophila*. Nature.

[bb0085] Gronke S., Clarke D.F., Broughton S., Andrews T.D., Partridge L. (2010). Molecular evolution and functional characterization of *Drosophila* insulin-like peptides. PLoS Genet..

[bb0265] Hirth F. (2010). *Drosophila* melanogaster in the study of human neurodegeneration. CNS Neruol. Disord. Drug Targets.

[bb0090] Hsu H.J., Drummond-Barbosa D. (2009). Insulin levels control female germline stem cell maintenance via the niche in *Drosophila*. Proc. Natl. Acad. Sci. USA.

[bb0095] Hsu H.J., LaFever L., Drummond-Barbosa D. (2008). Diet controls normal and tumorous germline stem cells via insulin-dependent and -independent mechanisms in *Drosophila*. Dev. Biol..

[bb0100] Hwangbo D.S., Gershman B., Tu M.P., Palmer M., Tatar M. (2004). *Drosophila* dFOXO controls lifespan and regulates insulin signalling in brain and fat body. Nature.

[bb0105] Ikeya T., Broughton S., Alic N., Grandison R., Partridge L. (2009). The endosymbiont Wolbachia increases insulin/IGF-like signalling in *Drosophila*. Proc. R. Soc. Lond. B Biol. Sci..

[bb0110] Jacobson J., Lambert A.J., Portero-Otin M., Pamplona R., Magwere T., Miwa S., Driege Y., Brand M.D., Partridge L. (2010). Biomarkers of aging in *Drosophila*. Aging Cell.

[bb0270] Jones M.A., Gargano J.W., Rhodenizer D., Martin I., Bhandari P., Grotewiel M. (2009). A forward genetic screen in *Drosophila* implicates insulin signaling in age-related locomotor impairment. Exp. Gerontol..

[bb0115] Kapahi P., Zid B.M., Harper T., Koslover D., Sapin V., Benzer S. (2004). Regulation of lifespan in *Drosophila* by modulation of genes in the TOR signaling pathway. Curr. Biol..

[bb0120] Karpac J., Hull-Thompson J., Falleur M., Jasper H. (2009). JNK signaling in insulin-producing cells is required for adaptive responses to stress in *Drosophila*. Aging Cell.

[bb0125] Kenyon C.J. (2010). The genetics of ageing. Nature.

[bb0130] LaFever L., Drummond-Barbosa D. (2005). Direct control of germline stem cell division and cyst growth by neural insulin in *Drosophila*. Science.

[bb0135] LaFever L., Feoktistov A., Hsu H.J., Drummond-Barbosa D. (2010). Specific roles of target of rapamycin in the control of stem cells and their progeny in the *Drosophila* ovary. Development.

[bb0140] Luong N., Davies C.R., Wessells R.J., Graham S.M., King M.T., Veech R., Bodmer R., Oldham S.M. (2006). Activated FOXO-mediated insulin resistance is blocked by reduction of TOR activity. Cell Metab..

[bb0145] Mair W., Dillin A. (2008). Aging and survival: the genetics of life span extension by dietary restriction. Annu. Rev. Biochem..

[bb0150] Mair W., Goymer P., Pletcher S., Partridge L. (2003). Demography of dietary restriction and death in *Drosophila*. Science.

[bb0155] Mair W., McLeod C.J., Wang L., Leanne Jones D. (2010). Dietary restriction enhances germline stem cell maintenance. Aging Cell.

[bb0160] McElwee J.J., Schuster E., Blanc E., Piper M.D., Thomas J.H., Patel D.S., Selman C., Withers D.J., Thornton J.M., Partridge L., Gems D. (2007). Evolutionary conservation of regulated longevity assurance mechanisms. Genome Biol..

[bb0165] Min K.T., Benzer S. (1997). Wolbachia, normally a symbiont of *Drosophila*, can be virulent, causing degeneration and early death. Proc. Natl. Acad. Sci. USA.

[bb0170] Min K.J., Yamamoto R., Buch S., Pankratz M., Tatar M. (2008). *Drosophila* lifespan control by dietary restriction independent of insulin-like signaling. Aging Cell.

[bb0175] Nielsen M.D., Luo X., Biteau B., Syverson K., Jasper H. (2008). 14-3-3 Epsilon antagonizes FoxO to control growth, apoptosis and longevity in *Drosophila*. Aging Cell.

[bb0180] Pan L., Chen S., Weng C., Call G., Zhu D., Tang H., Zhang N., Xie T. (2007). Stem cell aging is controlled both intrinsically and extrinsically in the *Drosophila* ovary. Cell Stem Cell.

[bb0185] Partridge L. (2010). The new biology of ageing. Philos. Trans. R. Soc. Lond. B Biol. Sci..

[bb0190] Piper M.D., Selman C., McElwee J.J., Partridge L. (2008). Separating cause from effect: how does insulin/IGF signalling control lifespan in worms, flies and mice?. J. Intern. Med..

[bb0195] Selman C., Lingard S., Choudhury A.I., Batterham R.L., Claret M., Clements M., Ramadani F., Okkenhaug K., Schuster E., Blanc E., Piper M.D., Al-Qassab H., Speakman J.R., Carmignac D., Robinson I.C., Thornton J.M., Gems D., Partridge L., Withers D.J. (2008). Evidence for lifespan extension and delayed age-related biomarkers in insulin receptor substrate 1 null mice. FASEB J..

[bb0200] Selman C., Tullet J.M., Wieser D., Irvine E., Lingard S.J., Choudhury A.I., Claret M., Al-Qassab H., Carmignac D., Ramadani F., Woods A., Robinson I.C., Schuster E., Batterham R.L., Kozma S.C., Thomas G., Carling D., Okkenhaug K., Thornton J.M., Partridge L., Gems D., Withers D.J. (2009). Ribosomal protein S6 kinase 1 signaling regulates mammalian life span. Science.

[bb0205] Slack C., Werz C., Wieser D., Alic N., Foley A., Stocker H., Withers D.J., Thornton J.M., Hafen E., Partridge L. (2010). Regulation of lifespan, metabolism, and stress responses by the *Drosophila* SH2B protein, Lnk. PLoS Genet..

[bb0210] Sun P., Quan Z., Zhang B., Wu T., Xi R. (2010). TSC1/2 tumour suppressor complex maintains *Drosophila* germline stem cells by preventing differentiation. Development.

[bb0215] Sykiotis G.P., Bohmann D. (2008). Keap1/Nrf2 signaling regulates oxidative stress tolerance and lifespan in *Drosophila*. Dev. Cell.

[bb0220] Tatar M., Kopelman A., Epstein D., Tu M.P., Yin C.M., Garofalo R.S. (2001). A mutant *Drosophila* insulin receptor homolog that extends life-span and impairs neuroendocrine function. Science.

[bb0225] Teleman A.A. (2010). Molecular mechanisms of metabolic regulation by insulin in *Drosophila*. Biochem. J..

[bb0230] Vellai T., Takacs-Vellai K., Zhang Y., Kovacs A.L., Orosz L., Muller F. (2003). Genetics: influence of TOR kinase on lifespan in *C. elegans*. Nature.

[bb0235] Wang M.C., Bohmann D., Jasper H. (2003). JNK signaling confers tolerance to oxidative stress and extends lifespan in *Drosophila*. Dev. Cell.

[bb0240] Wang M.C., Bohmann D., Jasper H. (2005). JNK extends life span and limits growth by antagonizing cellular and organism-wide responses to insulin signaling. Cell.

[bb0245] Wessells R.J., Fitzgerald E., Cypser J.R., Tatar M., Bodmer R. (2004). Insulin regulation of heart function in aging fruit flies. Nat. Genet..

[bb0250] Wessells R., Fitzgerald E., Piazza N., Ocorr K., Morley S., Davies C., Lim H.Y., Elmen L., Hayes M., Oldham S., Bodmer R. (2009). d4eBP acts downstream of both dTOR and dFoxo to modulate cardiac functional aging in *Drosophila*. Aging Cell.

[bb0255] Wong R., Piper M.D., Blanc E., Partridge L. (2008). Pitfalls of measuring feeding rate in the fruit fly *Drosophila melanogaster*. Nat. Meth..

[bb0260] Zid B.M., Rogers A.N., Katewa S.D., Vargas M.A., Kolipinski M.C., Lu T.A., Benzer S., Kapahi P. (2009). 4E-BP extends lifespan upon dietary restriction by enhancing mitochondrial activity in *Drosophila*. Cell.

